# Regional differences, repeated use, and costs of emergency medical services in Germany

**DOI:** 10.1007/s00063-024-01189-x

**Published:** 2024-09-25

**Authors:** Martin Roessler, Claudia Schulte, Christoph Bobeth, Isabelle Petrautzki, Laura Korthauer, Janosch Dahmen, Danny Wende, Christian Karagiannidis

**Affiliations:** 1 BARMER Institute for Health Care System Research, Berlin, Germany; 2 Faculty of Health, Department of Medicine, University Witten/Herdecke, Witten, Germany; 3 Lung centre Cologne-Merheim, ARDS and ECMO Centre, University Witten/Herdecke, Cologne, Germany

**Keywords:** Mortality, Hospitalization, Emergency medical department, Repeated users, Nursing home, Sterblichkeit, Hospitalisierung, Notaufnahme, Wiederholte Nutzer, Pflegeheim

## Abstract

**Background:**

Little is known about regional differences regarding the utilization and costs of emergency medical services (EMS) in Germany. Evidence on characteristics of repeated use of EMS is also scarce.

**Objectives:**

To compare German federal states regarding the utilization and costs of EMS and to analyze characteristics of repeated EMS use.

**Materials and methods:**

We used BARMER health insurance data on more than 1.4 million German EMS cases in 2022. We estimated EMS use rates (per 1000 inhabitants) and median reimbursements and costs by EMS type (ground transport with/without emergency physician (EP); helicopter emergency medical services), hospitalization status, and federal state. We applied Poisson regression to estimate incidence rate ratios (IRRs) with 95% confidence intervals (95% CI), capturing relationships between repeated use of EMS and individual characteristics, including care degree and income level.

**Results:**

Ground transport EMS use rates varied between federal states by more than 2.6-fold without EP (Bavaria: 84.6; Berlin: 223.2) and 2.1-fold with EP (Bremen: 19.1; Saxony: 41.3). Median reimbursement of ground transport with EP was 132% higher in Schleswig-Holstein (€ 1530) compared with Berlin (€ 660). Approximately one-third of all persons used EMS more than once and accounted for two-thirds of all EMS cases. Repeated EMS use was strongly related to care degree (IRR of care degree 5: 3084; 95% CI 3.012–3.158) and low income (IRR: 1.174; 95% CI 1.161–1.189).

**Conclusions:**

The substantial regional heterogeneity in terms of utilization and costs of EMS calls for a nationwide, consistent regulation of EMS in Germany. Additionally, (outpatient) primary nursing care of persons with severe health impairments and health literacy should be strengthened.

**Supplementary Information:**

The online version of this article (10.1007/s00063-024-01189-x) contains supplementary material, which is available to authorized users.

## Abstract (de)

**Hintergrund:**

Aktuell liegt wenig Evidenz zu regionalen Unterschieden in Hinblick auf die Nutzung und die Kosten des Rettungsdienstes (RD) in Deutschland sowie Charakteristika der wiederholen Nutzung des RD vor.

**Ziel der Arbeit:**

Vergleich der deutschen Bundesländer hinsichtlich der Nutzung und Kosten des RD sowie Analyse der wiederholten Nutzung des RD.

**Material und Methoden:**

Auf Basis von BARMER-Daten zu mehr als 1,4 Mio. RD-Fällen im Jahr 2022 wurden RD-Nutzungsraten (pro 1000 Einwohner) und Median-Vergütungen und -Kosten nach RD-Art (Bodentransport ohne/mit Notarzt [NA]; Luftrettung), Hospitalisierungsstatus und Bundesland geschätzt. Unter Anwendung von Poisson-Regressionen wurden „incidence rate ratios“ (IRR) mit 95 % Konfidenzintervallen (95 % KI) zur Abbildung von Zusammenhängen zwischen wiederholter RD-Nutzung und individuellen Charakteristika, darunter Pflegegrad und Einkommensniveau, geschätzt.

**Ergebnisse:**

Bodentransportraten variierten zwischen den Bundesländern um mehr als das 2,6-Fache ohne NA (Bayern: 84,6; Berlin: 223,2) und das 2,1-Fache mit NA (Bremen: 19,1; Sachsen: 41,3). Die Median-Vergütung für Bodentransporte mit NA lag in Schleswig-Holstein (1530 €) um 132 % höher als in Berlin (660 €). Rund ein Drittel aller Patienten nutzten den RD mehr als einmal und verursachten zwei Drittel aller RD-Fälle. Die wiederholte Nutzung des RD war stark mit dem Pflegegrad (IRR Pflegegrad 5: 3,084; 95 % KI 3,012–3,158) und niedrigem Einkommen (IRR: 1,174; 95 % KI 1,161–1,189) assoziiert.

**Diskussion:**

Die große regionale Heterogenität in der Nutzung und den Kosten des RD weist auf die Notwendigkeit einer einheitlichen Regulierung des RD in Deutschland hin. Zudem sollten die (ambulante) Pflege von Personen mit starken gesundheitlichen Einschränkungen und die Gesundheitskompetenz gestärkt werden.

Based on the recommendations of the government commission on hospital reform (German: “Regierungskommission für eine moderne und bedarfsgerechte Krankenhausversorgung”), the German government currently aims to reform the emergency care system. In this regard, one important aspect is the utilization of prehospital emergency medical services (EMS), which has increased substantially in recent years [16].

A previous analysis estimated that more than a quarter of all hospital cases were admitted via EMS in 2022 [14]. Most of these cases were characterized by very high age and up to 30% did not show characteristics indicating high severity. Other studies provided evidence that repeated/frequent users account for a relevant proportion of total EMS use [2, 3]. These findings may call for specific measures to avoid inefficiencies related to unnecessary utilization of EMS.

The legal framework, organization, execution, and funding of EMS are regulated differently in the 16 German federal states [11]. The responsibility for the EMS typically lies with the counties and independent cities, currently comprising nearly 300 independent EMS districts with around 240 dispatch centers featuring 13 different organizational forms. The EMS are operated by municipalities, charitable aid organizations, or private companies. This heterogeneity complicates planning across municipal boundaries and results in significant differences in the size, technical equipment, processes, performance, quality of services, and the qualifications of the personnel employed [13]. However, comprehensive data on the consequences of such heterogeneity are still missing.

Against that background, we used BARMER statutory health insurance data covering more than 10% of the German population in 2022 to explore regional heterogeneity regarding the use and costs of EMS. A special focus was placed on the prevalence and the characteristics of repeated users of EMS.

## Materials and methods

### Data

Our analysis was based on data from the statutory health insurance BARMER, covering about 8.7 million individuals from all over Germany in 2022.

We considered all EMS cases related to the transportation of persons insured with BARMER in 2022. EMS cases without transportation of persons (e.g., transportation of objects or empty runs) were not included in the analysis. Hospitalized EMS cases were identified via hospital admission at the day of EMS use. We used the main diagnosis of the respective hospital admission, coded according to the International Classification of Diseases, 10th Revision, German Modification (ICD-10-GM), to assess the main reason for inpatient treatment. The reimbursements of EMS uses were derived from the billing positions.

At the person level, we considered age (in years) at the time of EMS use, sex (male/female), and income level (low/medium/high/unknown). The income classification was based on household equivalence income and followed the social strata model of the German Institute for Economic Research (DIW) [9, 20].

We used information on individual care dependency as captured by care degrees (German: “Pflegegrade”). Care degrees range from 1–5, with a care degree of 3 or higher indicating severe or very severe impairment. We also considered whether a person was a nursing home resident at the time of EMS use.

We categorized each person’s place of residence according to the region type classification of the Bundesinstitut für Bau‑, Stadt- und Raumforschung (BBSR) as major city, urban, rural, or sparsely populated [1].

### EMS types

We differentiated between the following types of EMS based on billing data positions according to the German unified federal index of ambulance services (Bundeseinheitliches Positionsnummernverzeichnis für Krankentransportleistungen):

- Ground transport without presence of an emergency physician (EP),
- Ground transport with presence of an EP, and
- Use of helicopter emergency medical services (HEMS).

A more detailed, valid comparison of the use of different EMS types at the regional level was not possible due to heterogeneous coding and billing practices.

### Statistical analysis

We described categorical variables by absolute and relative frequencies and continuous variables by median and interquartile range (IQR). The frequencies of EMS use at the regional level were reported as rates (per 1000 inhabitants).

All estimates of EMS use proportions/rates, reimbursements, and costs were projected to the total German population (see supplementary material).

We modeled the number of EMS uses in 2022 at the person level within three different (sub)samples: 1) all EMS cases, 2) hospitalized EMS cases, 3) hospitalized EMS cases that received ground transport with EP or HEMS. We applied Poisson regression with the log. number of days the respective person was insured with BARMER in 2022 as offset to derive incidence rate ratios (IRRs) with 95% confidence intervals (95% CIs). When the link function is correctly specified, Poisson regression offers the advantage of providing consistent estimators of model coefficients, even if the outcome does not follow a Poisson distribution [7]. To account for potential deviation from equidispersion, we applied robust standard error estimators [7]. The significance level was set to 0.05.

Statistical analysis was conducted with R (version 3.6.3) [12].

## Results

### Case characteristics

We analyzed 1,450,278 EMS cases (Table 1). A total of 604,086 (41.7%) of these cases were hospitalized at the day of EMS use. While ground transport without EP was the most frequent EMS type in both hospitalized and nonhospitalized EMS cases, the proportions of ground transports with EP and HEMS was higher in hospitalized EMS cases. Median reimbursement was higher in hospitalized EMS cases (€ 700) than in nonhospitalized EMS cases (€ 400).

**Table 1:** Characteristics of included emergency medical cases cases

		Not hospitalized		Hospitalized	
Variable	Category	*n*/Median	%/(Q1; Q3)	*n*/Median	%/(Q1; Q3)
EMS type; *n*, %	Ground transport without EP	728,025	86.0	445,258	73.7
	Ground transport with EP	114,350	13.5	151,477	25.1
	HEMS	3817	0.5	7351	1.2
Reimbursement of EMS (1000 €); Median, (Q1; Q3)	–	0.4	(0.2; 0.7)	0.7	(0.4; 1.0)
Age; Median, (Q1; Q3)		76.0	(59.0;84.0)	78.0	(64.0;85.0)
Sex; *n*, %	Female	485,065	57.3	353,755	58.6
	Male	361,127	42.7	250,331	41.4
Nursing home resident; *n*, %	No	690,756	81.6	529,227	87.6
	Yes	155,436	18.4	74,859	12.4
Care degree; *n*, %	None	341,236	40.3	286,057	47.4
	1	24,992	3.0	22,190	3.7
	2	129,020	15.2	98,013	16.2
	3	176,643	20.9	107,707	17.8
	4	122,803	14.5	64,743	10.7
	5	51,498	6.1	25,376	4.2
Income level; *n*, %	High	165,497	19.6	114,768	19.0
	Low	267,965	31.7	193,194	32.0
	Medium	384,407	45.4	280,799	46.5
	Unknown	28,323	3.3	15,325	2.5
Region type;* n*, %	Major city	343,412	40.6	178,046	29.5
	Urban	272,686	32.2	225,195	37.3
	Rural	115,797	13.7	99,102	16.4
	Sparsely populated	114,297	13.5	101,743	16.8

*EMS* emergency medical services, *EP* emergency physician

Both hospitalized cases (IQR 64–85 years) and nonhospitalized cases (IQR 59–84 years) were characterized by high age. The groups showed similar distributions of sex and income levels.

Compared with hospitalized cases, both the proportions of nursing home residents and cases with strong health impairments (care degree ≥ 3) were higher in nonhospitalized cases.

### EMS use rates

**Fig. 1:**
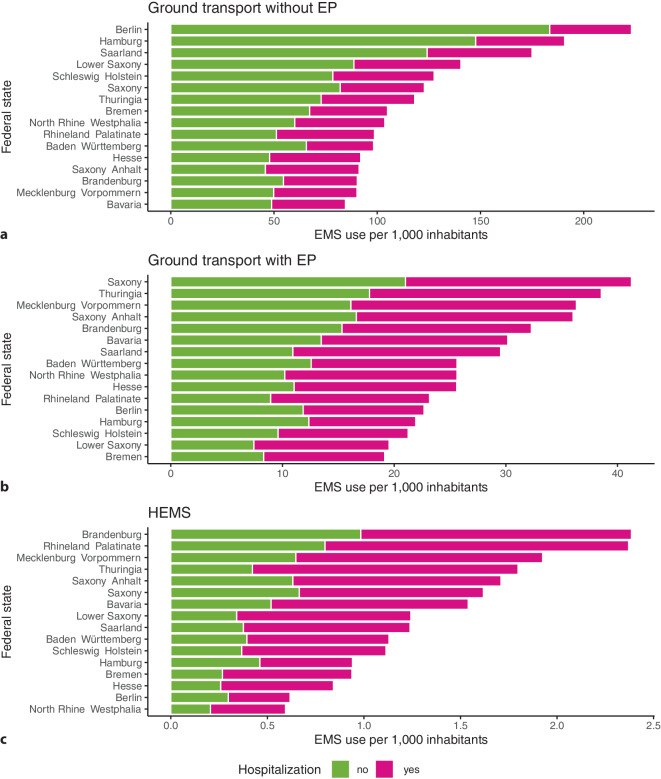
Frequency of emergency medical services (EMS) use per 1000 inhabitants by EMS type, hospitalization status, and German federal state. Scales are specific for each subfigure. *EP* emergency physician, *HEMS* helicopter emergency medical services

The rate of ground transport without EP varied from 84.6 in Bavaria to 223.2 in Berlin (Fig. 1a). Rates of ground transport with EP ranged from 19.1 in Bremen to 41.3 in Saxony (Fig. 1b). Per capita, HEMS were most often used in Brandenburg (rate: 2.4) and less often used in North Rhine–Westphalia (rate: 0.6) (Fig. 1c). The share of hospitalized cases was lowest for ground transport without EP and highest for HEMS.

### Reimbursement and costs of EMS use

**Fig. 2:**
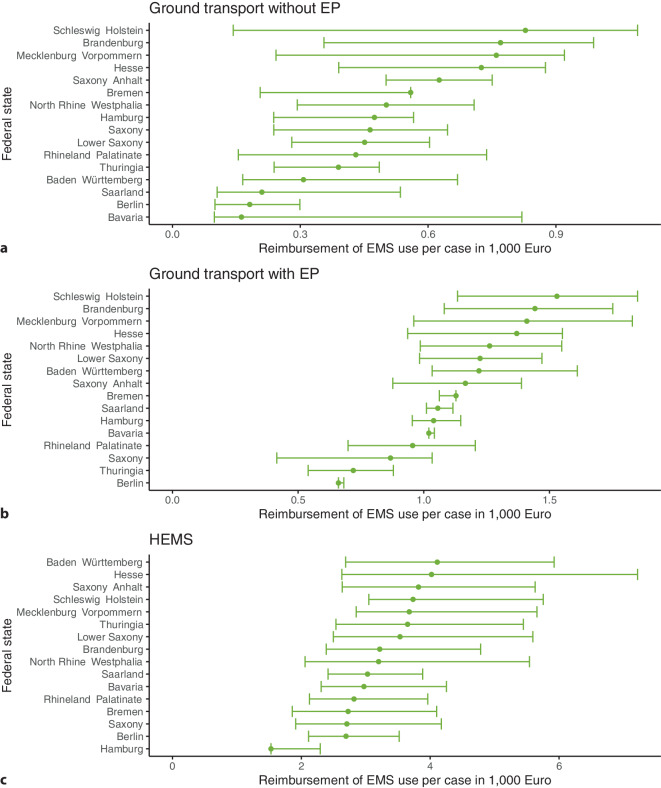
Reimbursement of emergency medical services (EMS) use per case in 1000 € (median and 1st and 3rd quartile) by EMS type and German federal state. Scales are specific for each subfigure. *EP* emergency physician, *HEMS* helicopter emergency medical services

The median reimbursement for ground transport without EP ranged from € 160 in Bavaria to € 830 in Schleswig–Holstein, with large IQRs (Fig. 2a). Involvement of an EP was associated with considerably higher median reimbursement, which ranged from € 660 in Berlin to € 1530 in Schleswig–Holstein (Fig. 2b). There was large variation in the median reimbursement for ground transport with EP also between the German city-states (Berlin: € 660; Hamburg: € 1040; Bremen: € 1130). HEMS was the most expensive EMS type in all federal states (range: € 1530 in Hamburg to € 4110 in Baden–Württemberg) (Fig. 2c).

Similar to reimbursements, we found huge regional heterogeneity in costs, even after adjustment for differences in age structure, number of inhabitants, and land area (Supplementary Figures S2, S3, and S4).

### Repeated EMS use

**Fig. 3:**
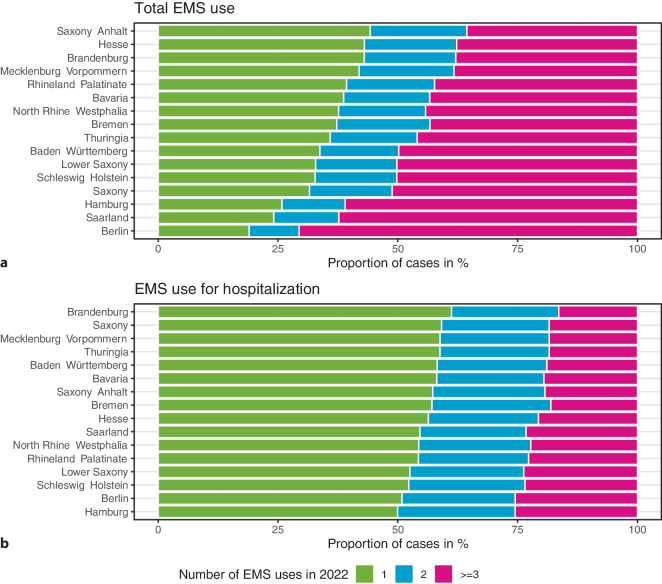
Proportion of emergency medical services (EMS) cases attributable to persons with 1, 2, or 3 or more EMS uses in 2022 by German federal state

Approximately 1/3 (33%) of all patients used EMS more than once in 2022. These patients accounted for approximately 2/3 (65%) of all EMS cases, with regional shares ranging from 55.8% in Saxony–Anhalt to 81.0% in Berlin (Fig. 3a). More than 1/3 (35.6%) of EMS cases in Saxony–Anhalt were caused by patients with at least 3 EMS cases in 2022. In Berlin, this was true for more than 2/3 (70.6%) of all EMS cases.

When considering EMS uses with subsequent hospitalizations only, the proportion of patients using EMS more than once in 2022 decreased to 23.4%. These patients accounted for 44.2% of all EMS uses with subsequent hospitalizations, with regional proportions raging between 38.8% in Brandenburg to 50.1% in Hamburg (Fig. 3b).

Frequent users with more than 10 hospitalizations using EMS in 2022 accounted for less than 1% of all cases. The proportion of inpatient main diagnoses of mental and behavioral disorders (ICD-10-GM: F*) ranged from 5.5% in patients using EMS for hospitalization only once to 38.2% in patients using EMS more than 10 times for hospitalization in 2022 (Supplementary Figure S5).

At the ICD‑3 level, I50: cardiac insufficiency (7.2%) and J44: Other chronic obstructive pulmonary disease (3.8%) accounted for 11% of all main diagnoses in patients with 3 or more hospitalizations using EMS (Supplementary Table S2).

### Characteristics of repeated EMS users

The average number of EMS cases per person in the samples considered in regression analyses was 1.98 for total EMS cases, 1.38 for hospitalized EMS cases, and 1.14 for hospitalized EMS cases receiving ground transport with EP or HEMS (Supplementary Table S3).

We found an inverted U‑shaped relationship between age and the rate of total EMS cases but not regarding repeated hospitalizations using EMS with EP or HEMS (Table 2). Men had a higher rate of EMS cases than women. Relative to persons with high income, persons with low income had a 17.4% higher rate of EMS cases (IRR 1.174; 95% CI 1.161–1.189) and a 12.9% higher rate of hospitalizations using EMS (IRR 1.129; 95% CI 1.123–1.135).

**Table 2:** Results of Poisson regressions^a^. Outcome: number of emergency medical services (EMS) uses in 2022

Sample	All EMS cases		Hospitalized EMS cases		Hospitalized EMS cases; ground transport with EP or HEMS only	
Variable	IRR	95% CI	IRR	95% CI	IRR	95% CI
Age: 50–59 (Ref.)	1	–	1	–	1	–
Age: < 18	0.767*	(0.753;0.782)	0.904*	(0.894;0.914)	0.955*	(0.942;0.968)
Age: 18–29	0.890*	(0.872;0.908)	0.987	(0.974;1.001)	1.018	(0.999;1.037)
Age: 30–39	0.939*	(0.916;0.962)	1.008	(0.993;1.022)	1.024*	(1.004;1.046)
Age: 40–49	0.967*	(0.944;0.991)	1.003	(0.990;1.015)	0.999	(0.985;1.014)
Age: 60–69	1.052*	(1.030;1.075)	1.020*	(1.012;1.029)	1.021*	(1.011;1.032)
Age: 70–79	1.027*	(1.006;1.048)	1.012*	(1.003;1.020)	1.015*	(1.006;1.025)
Age: 80–89	0.929*	(0.911;0.948)	0.982*	(0.974;0.990)	0.996	(0.987;1.006)
Age: 90+	0.822*	(0.804;0.840)	0.960*	(0.950;0.969)	1.045*	(1.031;1.060)
Sex: female (Ref.)	1	–	1	–	1	–
Sex: male	1.160*	(1.149;1.171)	1.083*	(1.078;1.087)	1.053*	(1.047;1.058)
Income level: high (Ref.)	1	–	1	–	1	–
Income level: low	1.174*	(1.161;1.189)	1.129*	(1.123;1.135)	1.117*	(1.109;1.125)
Income level: medium	1.084*	(1.072;1.096)	1.038*	(1.033;1.043)	1.025*	(1.018;1.031)
Income level: unknown	1.126*	(1.092;1.160)	1.084*	(1.070;1.098)	1.090*	(1.071;1.110)
Care degree: none (Ref.)	1	–	1	–	1	–
Care degree: 1	1.496*	(1.457;1.536)	1.208*	(1.195;1.221)	1.103*	(1.087;1.120)
Care degree: 2	1.893*	(1.864;1.922)	1.354*	(1.345;1.363)	1.151*	(1.139;1.162)
Care degree: 3	2.449*	(2.409;2.488)	1.517*	(1.507;1.527)	1.215*	(1.203;1.226)
Care degree: 4	2.864*	(2.813;2.917)	1.671*	(1.658;1.685)	1.294*	(1.278;1.309)
Care degree: 5	3.084*	(3.012;3.158)	1.766*	(1.747;1.786)	1.373*	(1.351;1.394)
Nursing home resident	1.200*	(1.182;1.218)	1.096*	(1.088;1.105)	1.026*	(1.016;1.037)
Region type: Major city (Ref.)	1	–	1	–	1	–
Region type: Urban	0.830*	(0.821;0.839)	0.981*	(0.977;0.986)	0.997	(0.991;1.004)
Region type: Rural	0.828*	(0.818;0.839)	0.984*	(0.978;0.990)	0.997	(0.990;1.005)
Region type: Sparsely populated	0.801*	(0.791;0.811)	0.969*	(0.963;0.974)	0.993	(0.986;1.001)
(Intercept)	0.004*	(0.004;0.004)	0.003*	(0.003;0.003)	0.003*	(0.003;0.003)
*n*	730,777	–	436,872	–	139,734	–

*IRR* Incidence rate ratio, *CI* confidence interval

*significant at the 5% level

^a^Poisson regression with robust standard errors; the log. number of days a person was insured with BARMER in 2022 was used as offset

Relative to persons without care degree, the rate of total EMS cases was more than 200% higher (IRR 3.084; 95% CI 3.012–3.158) and the rate of hospitalizations using EMS was 76.6% higher (IRR 1.766; 95% CI 1.747–1.786) in persons with care degree 5. Similar associations were found with care degrees 3 and 4.

Nursing home residents had a 20% higher rate of total EMS cases (IRR 1.2; 95% CI 1.182–1.218) and a 9.6% higher hospitalization rate using EMS (IRR 1.096; 95% CI 1.088–1.105).

We found that repeated EMS use was less common in sparsely populated areas than in major cities (IRR 0.801; 95% CI 0.791–0.811). There was no association between repeated EMS use and region type when considering hospitalized EMS cases receiving ground transport with EP or HEMS only.

## Discussion

Our analysis revealed substantial regional heterogeneity regarding the utilization and costs of EMS in Germany. Use rates of EMS without EP varied between German federal states by more than 2.6-fold (Bavaria: 84.6; Berlin: 223.2) and use rates of EMS with EP by more than 2.1-fold (Bremen: 19.1; Saxony: 41.3). The median reimbursement of ground transport with EP was 132% higher in Schleswig–Holstein (€ 1530) compared with Berlin (€ 660). These findings reflect the absence of a nationwide, consistent regulation of EMS, which results in huge differences in utilization and cost structures between German federal states. Regulatory heterogeneity between federal states and even between lower-level regional units, such as counties and municipalities, may induce relevant procedural and financial inefficiencies that place an avoidable burden on the German health system.

We estimated that 1/3 of all persons used EMS more than once in 2022. Those repeated users accounted for 2/3 of all EMS cases. Repeated users were also represented in the subgroup of hospitalized EMS cases, where 23.4% of the persons accounted for 44.2% of all cases. Our regression results indicated that particularly care-dependent persons showed a high intensity of EMS use. Common diagnoses in patients with repeated or frequent use of EMS were heart failure, chronic obstructive pulmonary disease (COPD) and mental and behavioral disorders. Despite the burden of the diseases, this points towards innovative therapy and nursing approaches to avoid hospitalization, i.e., early treatment at home, telemonitoring, or early interventions [6, 18].

Another characteristic associated with the repeated use of EMS was the individual’s socioeconomic status, here measured by income. Adjusting for covariates, individuals with low income had a 17.4% higher EMS use rate compared with individuals with high income. This result may be driven by the association between socioeconomic status and health status, which implies that lower income levels tend to be related to higher morbidity [10, 15]. This association, in turn, is closely related to the individuals’ health literacy [8, 19]. Strengthening health literacy and implementing preventive emergency services units, therefore, may also contribute to a more effective and efficient use of health care services, including EMS [4, 5].

### Strengths and limitations

A main strength of our analysis is the use of data on more than 1.4 million EMS cases in 2022 from all German federal states. These data provided a valid and comprehensive basis for exploration and regional comparison of EMS use and costs. By using data from different health care sectors, we were able to differentiate between hospitalized and nonhospitalized EMS cases and to explore characteristics associated with the repeated use of EMS.

A limitation of the data used in this analysis is the lack of valid information on reasons for EMS use. Accordingly, we could not explore why individuals called the emergency number or for what reasons they were transported when there was no subsequent hospitalization.

There was also no complete and valid information on the place of operation coded in EMS billing data. We therefore used the individual’s place of residence to assign EMS use to one of the 16 German federal states. Consequently, there likely was some spatial misclassification, which may bias estimates of regional EMS use rates, reimbursements, and costs. However, elderly, care-dependent individuals are usually subject to limited mobility. Particularly for these individuals, which account for a large share of total EMS use, the region of residence is generally expected to coincide with the region of EMS operation.

For some individuals, the income level could not be determined. This may have caused bias in estimators of IRRs capturing relationships between repeated EMS use and socioeconomic status. However, the magnitude of such bias will likely be low since income data were missing for only 3% of all cases.

The data used were limited to a single health insurance fund covering approximately 10% of the German population. Despite the lack of a complete survey, we consider the data to be representative due to the large amount of data and the comprehensive regional, age, and gender distribution.

Finally, regional differences in coding practices may have induced distortions in our analysis. We can therefore not exclude that some of the regional variation revealed in our analysis is not related to the provision of EMS but to billing characteristics.

## Conclusions and suggested solutions

The huge regional heterogeneity in terms of emergency medical services (EMS) use and costs and the prominent role of repeated EMS users revealed in this study indicate the need for an urgent reform of the German emergency medical care, in particular EMS.

Some solutions that may be addressed within the upcoming reforms include:

- The current reform of the German emergency care system should also include a reform of the EMS system.
- Inefficiencies related to regional heterogeneity should be eradicated through the implementation of nationwide rules and standards. To achieve this objective, the regulation of the EMS system should become part of the Fifth Book of the German Social Code (SGB V).
- For better quality assurance and public health research, the availability of standardized datasets, such as the Minimal Emergency Data Set (MIND), should be legally mandated for all EMS patients and consolidated in emergency medical registries.
- Primary care for elderly and care-dependent people should be strengthened, e.g., by community health nursing.
- The potentials of innovative approaches for avoidance of hospitalization should be exploited, especially in care-dependent individuals and patients with chronic diseases such as heart failure or chronic obstructive pulmonary disease (COPD).

## Supplementary Information

Results of additional statistical analyses

## Data Availability

The data used in this study cannot be made available in the manuscript, the supplemental files, or in a public repository due to German data protection laws (*Bundesdatenschutzgesetz*). Therefore, they are stored on a secure drive at Barmer insurance to facilitate replication of the results. Generally, access to data of statutory health insurance funds for research purposes is possible only under the conditions defined in German Social Law (SGB V § 287). Requests for data access can be sent as a formal proposal specifying the recipient and purpose of the data transfer to the appropriate data protection agency. Access to the data used in this study can only be provided to external parties under the conditions of the cooperation contract of this research project and after written approval by Barmer insurance.

## Declarations

For this article no studies with human participants or animals were performed by any of the authors. All studies mentioned were in accordance with the ethical standards indicated in each case. Approval of this study by an ethics committee was not required due to the use of fully pseudonymized, secondary data. Data preparation and analysis followed the Guidelines and Recommendations for Good Practice of Secondary Data Analysis [17]. The study adheres to all relevant legal regulations, including the General Data Protection Regulation (GDPR), and national and international guidelines, including the Declaration of Helsinki.
