# Supplementary material for: Regional differences, repeated use, and costs of emergency medical services in Germany
Source: Med Klin Intensivmed Notfmed. 2024 Sep 25;120(7):576–84. doi: 10.1007/s00063-024-01189-x (PMC12504377; doi:10.1007/s00063-024-01189-x)
Supplement: Supplementary file 1 — Results of additional statistical analyses [file 63_2024_1189_MOESM1_ESM.pdf]

Supplementary material
Regional heterogeneity, (repeated) use, and costs of emergency medical services in Germany

Martin Roessler[a], Claudia Schulte[a], Christoph Bobeth[a], Isabelle Petrautzki[a], Laura Korthauer[a], Janosch Dahmen[b], Danny Wende[a], Christian Karagiannidis[c]

[a] BARMER Institute for Health Care System Research, Axel-Springer-Str. 44, 10969 Berlin
[b] Faculty of Health, Department of Medicine, University Witten/Herdecke
[c] Lung centre Colgone-Merheim, ARDS and ECMO Centre, University Witten/Herdecke

Projection of statistics to the total German population

All estimates of rates, proportions, reimbursements, and costs were projected to the total German population using inverse market shares of BARMER in 2022 by

- 400 German counties (“Kreise and kreisfreie Städte”) ,
- sex (male/female),
- age groups (age years: 0-2, 3-5, 6-9, 10-14, 15-17, 18-19, 20-24, 25-29, 30-34, 35-39, 40-44, 45-49, 50-54, 55-59, 60-64, 65-74, 75+).

Market shares were calculated as the number of persons insured with BARMER relative to the total population in the considered region-sex-age-stratum at December 31, 2022.

Shares of patient transport service ambulance in cases and costs of EMS cases receiving ground transport without EP

The proportion of cases and costs in EMS cases receiving ground transport without EP, which was attributable to the use of patient transport service ambulance (PTS, German: Krankentransportwagen) varied substantially between German federal states (Table S1). However, the validity of these estimated proportions may be limited due to regional differences in coding and billing practices. Hence, these proportions should be interpreted with caution.

Table S1: Shares of patient transport service ambulance in cases and costs of EMS cases receiving ground transport without EP

Federal state	Share of cases in %	Share of costs in %
Saxony-Anhalt	28,3	19,3
Hesse	29,9	14,1
Brandenburg	30,6	16,6
Bremen	37,1	19,6
Mecklenburg-Vorpommern	40,5	21,4
Thuringia	42,3	30,8
Lower Saxony	44,6	32,4
North Rhine-Westphalia	46,9	32,7
Schleswig-Holstein	48,8	20,6
Rhineland-Palatinate	50,7	23,0
Hamburg	53,2	37,0
Saxony	54,7	38,7
Baden-Württemberg	60,8	36,2
Bavaria	70,5	32,4
Saarland	71,3	49,1
Berlin	74,6	57,7

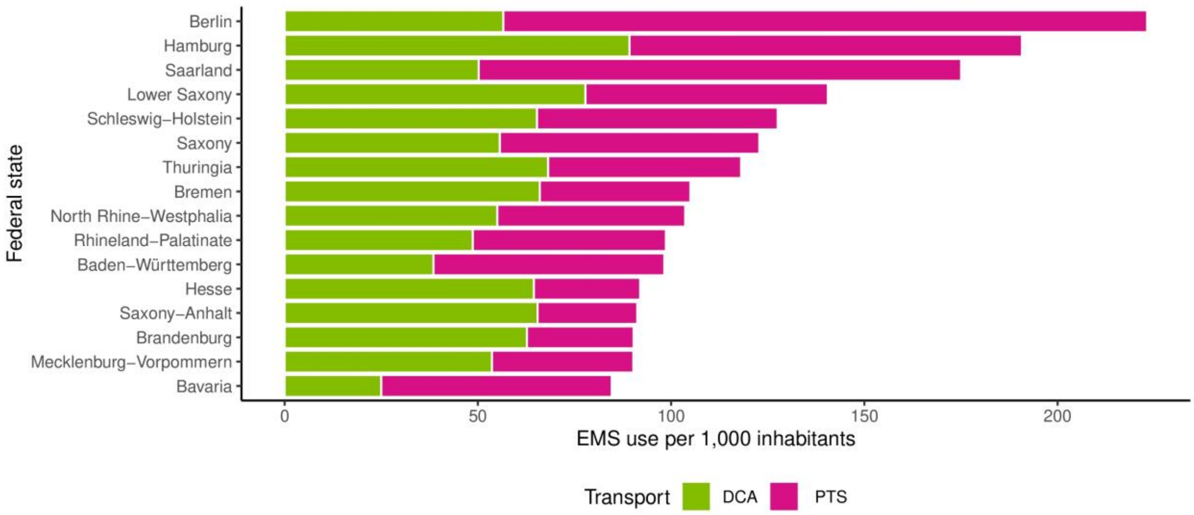

Figure S1: EMS use rates for ground transport without emergency physician by federal state and vehicle
DCA = Double crewed ambulance (German: Rettungswagen); PTS = patient transport service ambulance (German: Krankentransportwagen)

Age standardized costs of EMS use per inhabitant

For direct age standardization, we calculated the average costs per inhabitant within federal states and age groups (see above). We then used the share of these age groups in the total German population to project the average costs per inhabitant of each federal state to the total population.

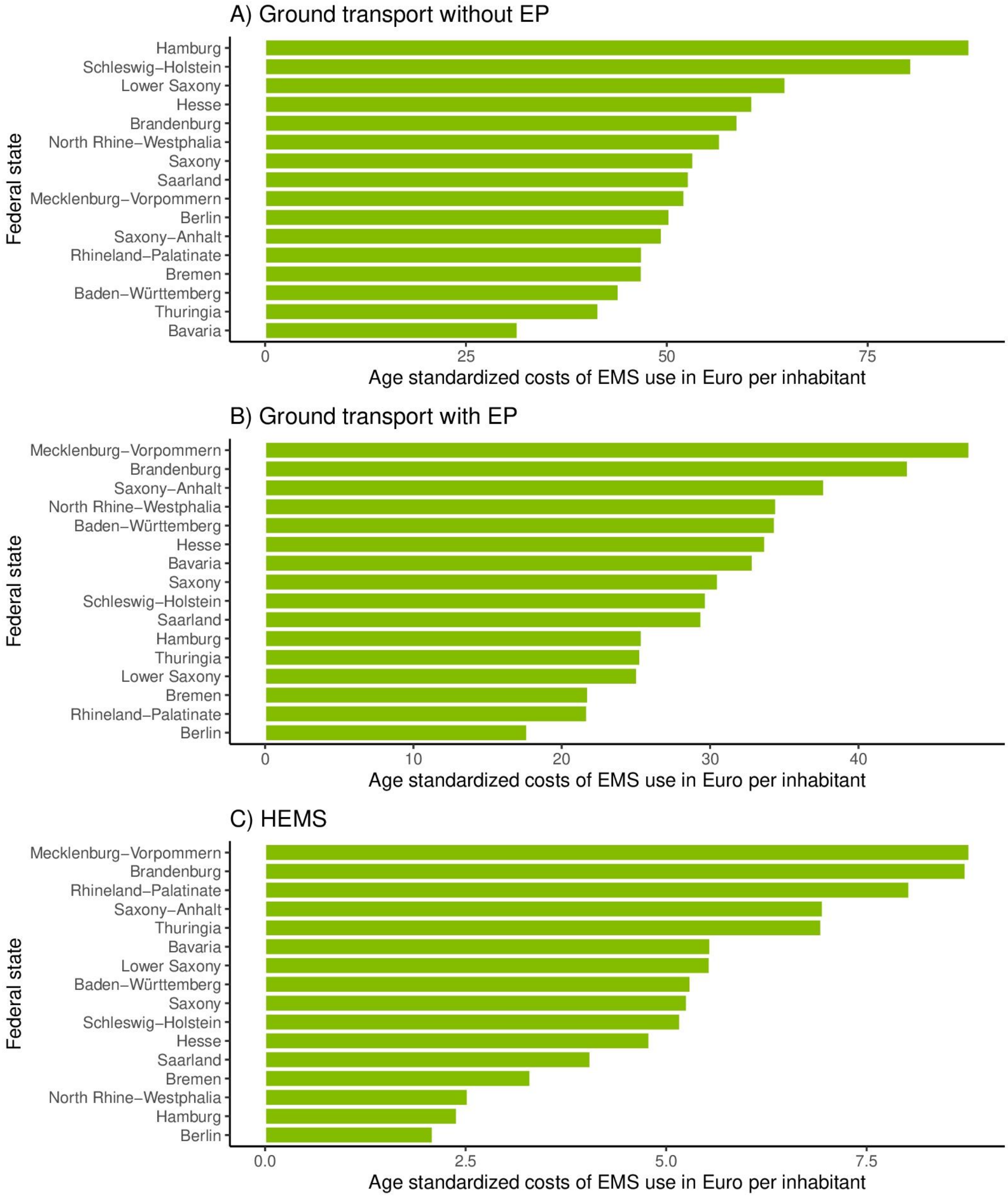

Figure S2: Age standardized costs of EMS use in Euro per inhabitant

Calculation of standardized cost rates

Standardization of total costs at the federal state level was based on a Cobb-Douglas type cost function:

$$\log C_i = \beta_0 + \beta_1 \log I_i + \beta_2 \log A_i + u_i,$$

where C_i denotes age standardized total costs, I_i denotes the number of inhabitants, A_i denotes land area and u_i, $(E[u_i|I_i, A_i] = 0)$ is the residual of the federal state i (see Figure S3 for scatter plots of these variables).

Given parameter estimates $\hat{\beta}_0$, $\hat{\beta}_1$, and $\hat{\beta}_2$ derived via linear regression, the standardized cost rate of a specific federal state was calculated as

$$SCR_i = \frac{C_i}{\hat{C}_i} = \frac{C_i}{\exp(\hat{\beta}_0 + \hat{\beta}_1 \log I_i + \hat{\beta}_2 \log A_i)} = \frac{C_i}{\exp(\hat{\beta}_0) I_i^{\hat{\beta}_1} A_i^{\hat{\beta}_2}}.$$

Accordingly, standardized cost rates above 1 indicate higher than expected costs whereas standardized cost rates below 1 indicate lower than expected costs (Figure S4).

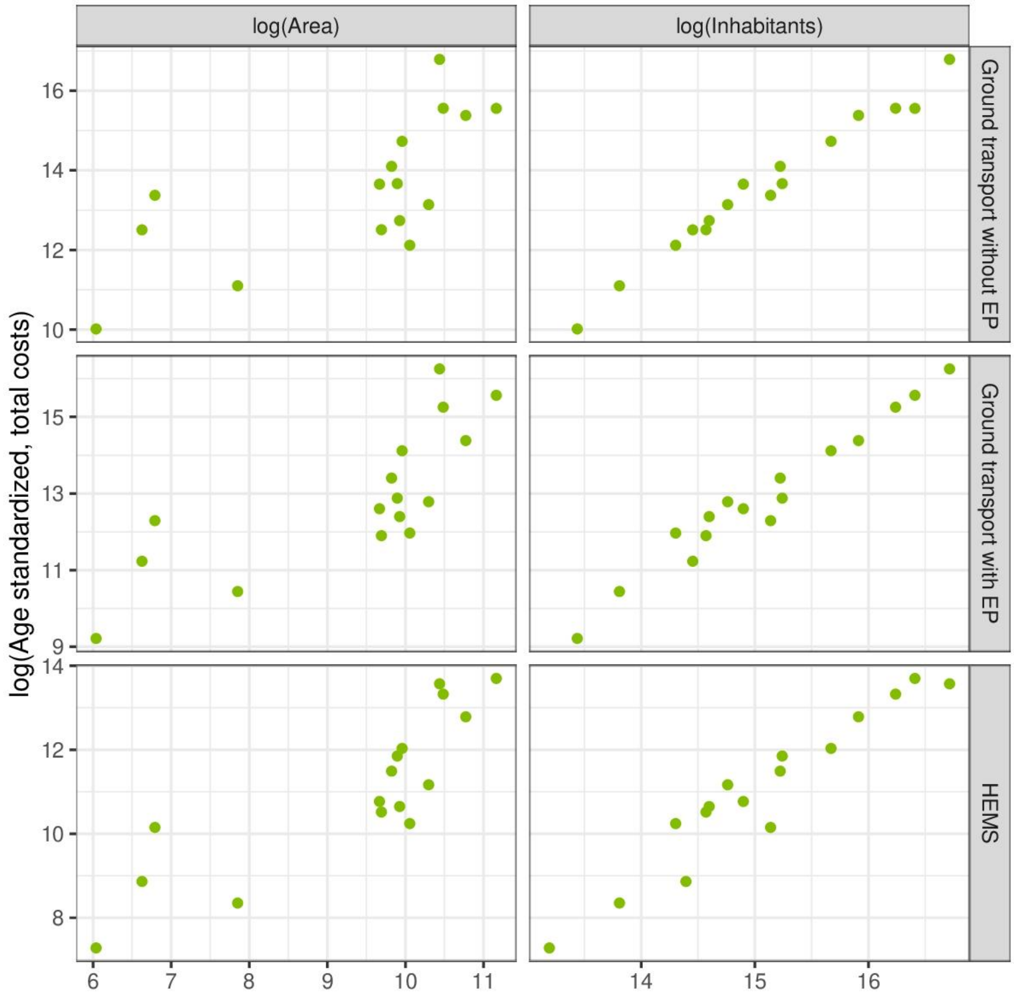

Figure S3: Relationships between age standardized total costs, area, and number of inhabitants by type of EMS at the federal state level

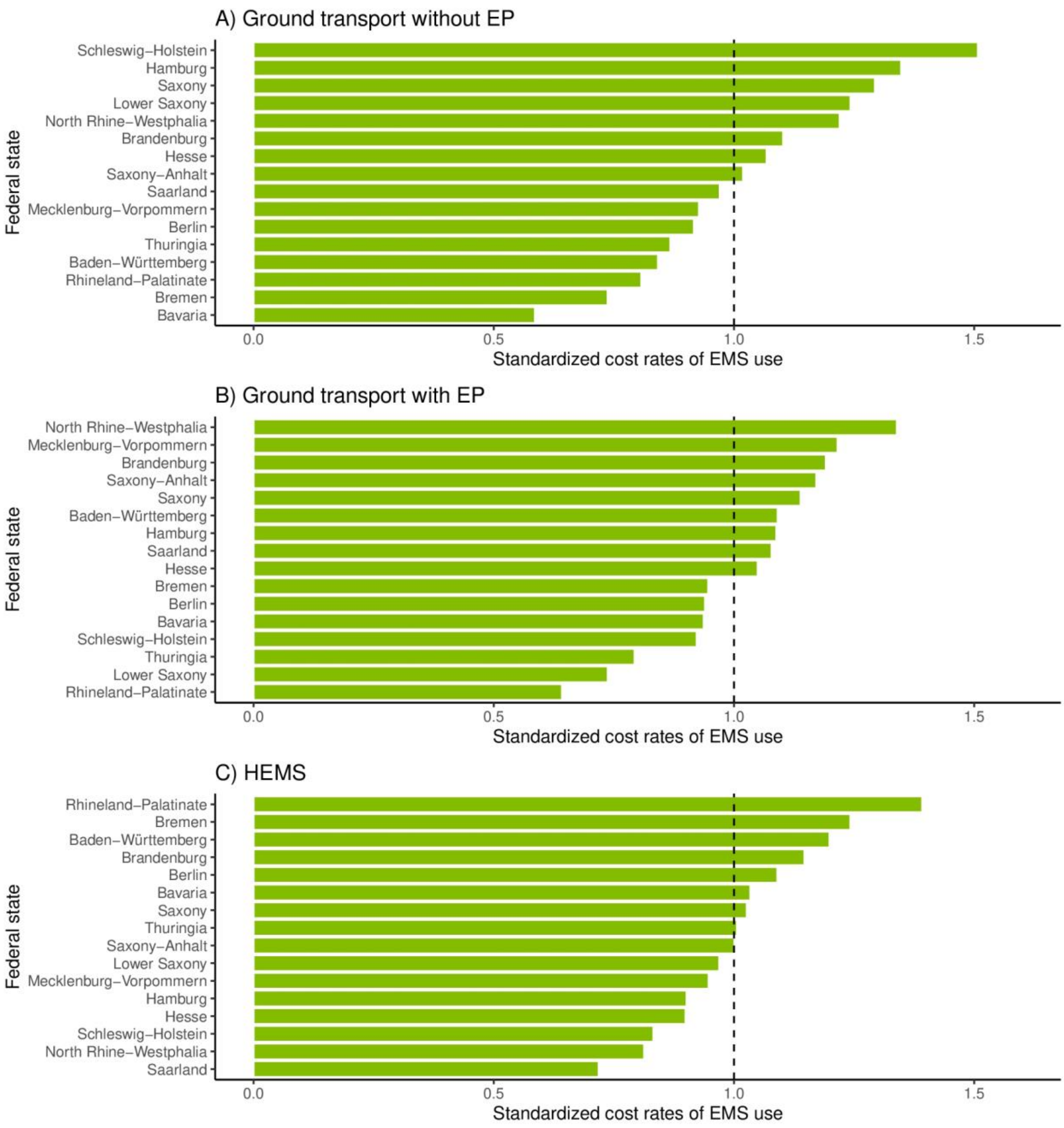

Figure S4: Standardized cost rates by EMS type and federal state.

Repeated EMS use

Table S2: Top-10 main diagnoses in hospitalized EMS users by number of hospitalizations using EMS in 2022

Rank	Number of EMS uses: 1	Number of EMS uses: 2	Number of EMS uses: >=3
1	3.8 %: S72 Fracture of the femur	6.0 %: I50 Heart failure	7.2 %: I50 Heart failure
2	3.7 %: I50 Heart failure	3.8 %: I63 Cerebral infarction	3.8 %: J44 Other chronic obstructive pulmonary disease
3	3.7 %: I63 Cerebral infarction	3.7 %: S72 Fracture of the femur	3.5 %: F10 Mental and behavioral disorders caused by alcohol
4	3.4 %: S06 Intracranial injury	2.6 %: E86 Volume deficiency	3.1 %: N39 Other diseases of the urinary system
5	3.1 %: I21 Acute myocardial infarction	2.6 %: I21 Acute myocardial infarction	2.8 %: E86 Volume deficiency
6	2.1 %: I48 Atrial fibrillation and flutter	2.5 %: S06 Intracranial injury	2.5 %: I63 Cerebral infarction
7	2.0 %: R55 Syncope and collapse	2.5 %: N39 Other diseases of the urinary system	2.4 %: S72 Fracture of the femur
8	2.0 %: I10 Essential (primary) hypertension	2.4 %: J44 Other chronic obstructive pulmonary disease	2.3 %: S06 Intracranial injury
9	1.7 %: J18 Pneumonia, pathogen unspecified	2.1 %: J18 Pneumonia, pathogen unspecified	2.2 %: J18 Pneumonia, pathogen unspecified
10	1.7 %: E86 Volume deficiency	1.8 %: I48 Atrial fibrillation and flutter	1.9 %: G40 Epilepsy

Table S3: Summary of repeated EMS use by (sub-)sample

Group	Persons	Cases	Cases/Person
Total EMS cases	730,777	1,450,278	1.98
Hospitalized EMS cases	436,872	604,086	1.38
Hospitalized EMS cases; ground transport with EP or HEMS only	139,734	158,828	1.14

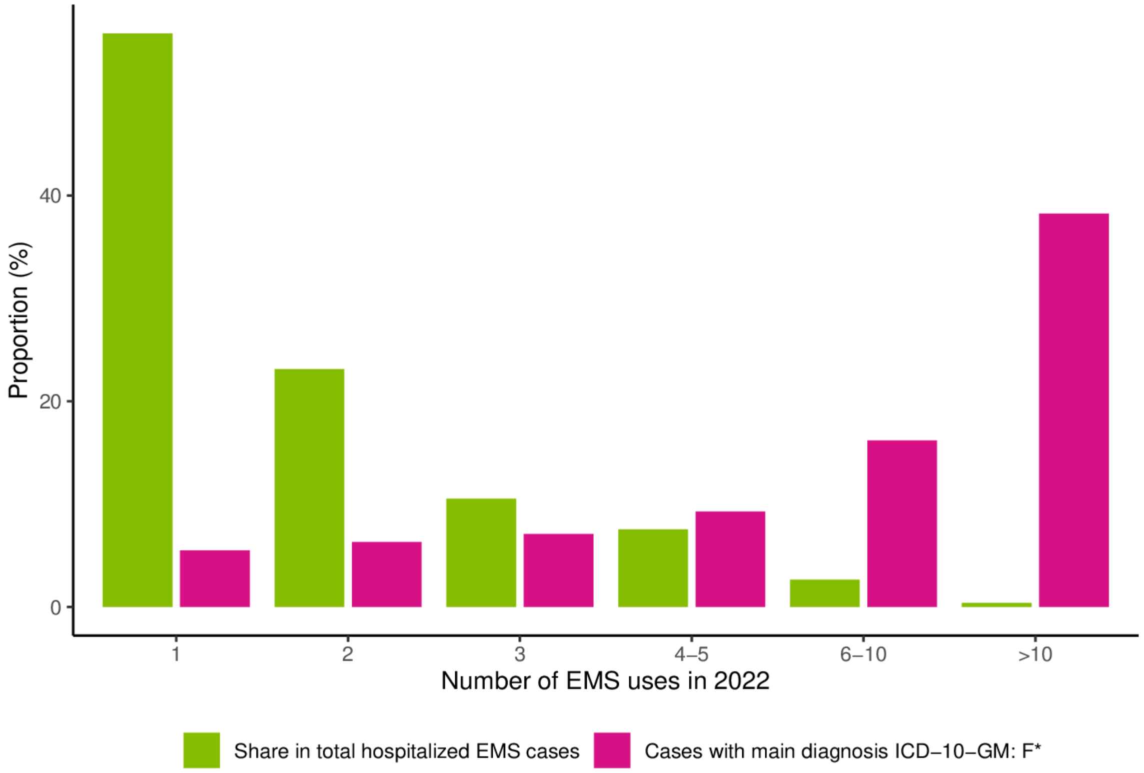

Figure S5: Share of hospitalized EMS cases in total EMS cases and share of hospitalized EMS cases with main diagnosis of mental and behavioral disorders (ICD-10-GM: F*) by number of EMS uses in 2022
